# The Role of Self-esteem and Fear of Negative Evaluation in Compulsive Buying

**DOI:** 10.3389/fpsyt.2017.00074

**Published:** 2017-05-02

**Authors:** Roberta Biolcati

**Affiliations:** ^1^Department of Education Studies, University of Bologna, Bologna, Italy

**Keywords:** compulsive buying, contingent self-esteem, fear of negative evaluation, gender differences, adult population

## Abstract

Compulsive buying is a relatively new addictive disorder that interferes with everyday functioning and may result in serious psychological and financial problems (1). A very few data are currently available regarding this behavioral addiction. This study investigated gender differences in the relationships between contingent self-esteem (CSE), fear of negative evaluation (FNE), and compulsive buying. Participants included 240 Italian adults (170 females, M age = 33.80) who responded to self-report questionnaires. The results showed that women scored higher on CSE and FNE scales than men. No gender differences were found in compulsive buying tendencies. CSE and FNE were positively related to CB. Furthermore, structural equation modeling confirmed the evidence on CSE as a strong predictor of CB for both genders. Interestingly, FNE seems to play a mediating role between CSE and compulsive buying behaviors only for women. These findings highlight the importance of studying self-esteem in compulsive buying tendencies to inquire more deeply into the underlying mechanisms of some compulsive behaviors.

## Introduction

Compulsive shopping is a dysfunctional form of excessive and pervading buying (2–4). The consumer experiences an unstoppable, chronic, and repeated impulse to go shopping and spend money and loses control of this activity (5). The psychosocial consequences of compulsive buying (or shopping) behavior can be summarized as: a great amount of debts, inability to meet payments, legal and financial problems, self-criticism, guilt, and personal suffering [e.g., Ref. (6, 7)].

Results from a meta-analysis of 40 studies from 16 countries reported a prevalence of compulsive buying of 4.9% in the adult population (8) and the age of onset appears to be around 30 (1, 9).

Most clinical studies report that women are much more likely to become compulsive buyers than men [e.g., Ref. (10)] but, in the last decade, scholars have observed no significant differences based on gender (11–14).

Compulsive buying is primarily motivated by the relief of anxiety, stress, and unpleasant emotions (15–17).

Dysfunctional consumer behavior is often enacted for some perceived psychological advantages (18). In particular, the desire to improve emotions related to *self-esteem* is a prominent motivation that can predict this form of behavioral addiction (19, 20). Ertelt et al. (21) showed that compulsive buyers suffer from low self-esteem and seek approval from others to compensate it.

The “consumption of objects” may be a coping response to unpleasant emotions deriving from insecurity about individuals’ selves and identities and disappointing self-evaluations (16, 22). Some people may use shopping to improve their appearance, self-confidence, consideration by others, and social relationships (23). Material possessions respond illusorily to the inner security need to compensate feelings of low self-esteem and, at the same time, current consumer culture suggests that a sense of self-confidence and well-being can be purchased. Therefore, the “consumption of objects” may be both a symptom of insecurities and a “coping strategy” to offer relief from problems and to satisfy certain needs (24–26).

Some products play a peculiar role for compulsive buyers due to the emotional and social advantages they offer (27). By buying objects, compulsive shoppers try to address to an “ideal self,” improve their social image and express themselves (20), such that perceived self-esteem benefits represent a direct predictor of compulsive buying (18).

### Contingent Self-esteem (CSE)

At the same line, Roberts et al. (28), in accordance with the Self-Determination Theory (29, 30), argue that self-esteem varies along a continuum that ranges from true self-esteem to CSE. True self-esteem is a more stable individual characteristic that is not dependent upon meeting external standards or others’ approval. At the other end of the continuum is CSE, where one’s self-esteem is dependent on matching some self-imposed or external standard or meeting some objective. Individuals with a high CSE score may base their self-worth on their physical attractiveness, social conditions, or job performance (31, 32).

In people with high CSE, searching and maintaining positive self-definition becomes the main orientation, expressed also through their behaviors. They direct their efforts to appearing worthy to others and feel that they must achieve something in order to justify their positive feelings toward themselves (29, 33, 34).

Very little research has focused on gender differences in CSE. The few studies indicated that women report greater CSE compared to men (32, 35).

Previous studies have investigated the role of CSE in relation to body image and body dissatisfaction [e.g., Ref. (36)] or in relation to mental health and alcohol use [e.g., Ref. (37, 38)]. To our knowledge, only one previous study (28) has investigated the predictive role of CSE on compulsive buying behavior.

### The Fear of Others’ Judgment

In addition, compulsive shoppers, especially women, give more importance to disapproval and are more likely to engage in social comparison than others (39). Indeed, compulsive shoppers are more inclined to base their instable self-esteem on the judgment of others and often purchase things to impress other people (40, 41).

Roberts et al. (28) proposed the mediation role of the fear of negative evaluation (FNE) on CSE and compulsive buying. They confirmed the importance of CSE to predict compulsive buying through the mediation of FNE, in particular for the adult population. Specifically, FNE is defined as a personal trait consisting in apprehension about others’ evaluations, distress over their negative evaluations, and the desire to seek social approval and avoid disapproval (42). To date, it has been considered a cognitive and emotional risk factor for social anxiety [e.g., Ref. (43)] and eating disorders, especially for women [e.g., Ref. (44, 45)]. A previous study investigated the FNE as a potential mediator between social anxiety and disordered eating in females (46). Generally, studies reported that women scored significantly higher on the FNE scale than men [e.g., Ref. (47)]. However, much of the research on women’s social comparison and fear of evaluation has been in the field of body image and attractiveness (48) and does not include compulsive buying.

The main objective of this study is to deepen our knowledge of compulsive buying and gender differences in its underlying mechanisms. Specifically, the aim is to examine if and how CSE predicts compulsive buying through the mediation of FNE in men and women.

### Hypotheses

Our first hypothesis concerns the differences between men and women in the variables analyzed. Specifically, in line with the literature previously described, we predict that women will score higher on CB, CSE, and FNE than men (H1).

**Figure 1:**
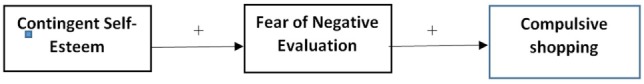
**The theoretical model**.

Our second hypothesis is summarized in the theoretical model presented in Figure 1. More specifically, we expect that, in both men and women, CSE predicts CB (H2). As shown by the literature, CSE is an individual characteristic referring to feelings about oneself that are dependent on matching some standard or living up to others’ expectations; this fragile sense of self is a risk factor for CB in both genders.

Our third hypothesis is that FNE fully or partially mediates the relationship between CSE and CB (H3). The prediction is expected to be stronger for women, on account of their greater sensitivity to others’ judgments, their higher social self-consciousness, and their supposed greater FNE and punishment (49).

## Materials and Methods

### Participants

Two hundred forty subjects [M = 29.2% (70); F = 70.8 (170)] aged between 18 and 61 (mean age = 33.80, SD = 10.61) were recruited online. As regards marital status, 148 (61.7%) were unmarried, 75 (31.2%) were married or living with a partner, 16 (6.7%) were divorced or separated, and 1 (0.4%) was widowed.

Sixteen participants (6.7%) had a primary school or a junior high certificate, 83 (34.6%) had a high school diploma, 72 (30.0%) had graduated in the first cycle degree, 50 (20.8%) had graduated in the second cycle degree, and 19 (7.9%) had a specialization or Ph.D.

With regards socioeconomic status, 44.6% declared they had an annual income of less than 15,000 Euros, 37.9% between 15,000 and 30,000, 9.6% between 30,000 and 45,000, 3.3% between 45,000 and 60,000, and 1.3% more than 60,000.

### Procedure

An online survey was constructed using LimeSurvey software (http://www.limesurvey.org). Participants were contacted through social networks (e.g., Facebook) and by e-mail spreading. Participants were asked to answer some questions on purchasing behavior. No personal identifying information was collected. As standard procedure for minimal-risk online survey, the study waived documentation of informed consent, by permitting continued participation to signal consent. The Ethics Commission of the institution where the author work approved this survey, which was conducted in agreement with the ethical norms laid down by the Italian National Psychological Association.

### Measures

The questionnaire included a first section regarding age, sex, education level, income, time spent in shopping, average amount spent per shopping trip, credit card usage, and influence of advertising on purchases.

#### Compulsive Buying

A questionnaire including 13 statements [compulsive buying scale (CBS); (50)], evaluated on a 5-point scale of disagreement/agreement, in its Italian version (5), was used to measure compulsive buying. Sample items include the following: “When I have money, I cannot help but spend part or all of it”; “At times, I have felt somewhat guilty after buying a product, because it seemed unreasonable.” Cronbach’s α = 0.91.

#### Contingent Self-esteem

An 8-item scale (31) was used to measure CSE (evaluated on a 5-point scale of disagreement/agreement). Although the original scale contains 15 items, we used the version tested by Roberts et al. (28) measuring self-esteem contingencies with regard to gaining others’ approval. Sample items include the following: “My overall feelings about myself are heavily influenced by how much other people like and accept me” and “My overall feelings about myself are heavily influenced by how good I look.” Cronbach’s α = 0.81.

#### Fear of Negative Evaluation

The brief version of the FNE (51) was used to measure fears of being observed and evaluated by others. We deleted four items according to the version proposed by Roberts et al. (28). Sample items include the following: “I am afraid others will not approve of me” “When I am talking to someone, I worry about what they may be thinking of me.” Subjects rate their agreement with statements on a 5-point scale, where 1 = not very characteristic of me to 5 = very characteristic of me. Cronbach’s α = 0.92.

### Analytical Procedures

Data were analyzed using SPSS version 18 (Chi-square and ANOVA analysis) to test Hypothesis 1. Subsequently, the mediation model (Hypothesis 2 and Hypothesis 3) was tested using multiple-step regression analysis by the PROCESS macro for SPSS (52). In particular, we used the so-called “Model 4” (simple mediation model) methodology.

## Results

### Compulsive Buying

Preliminarily, in order to explore clinical implications, we examined the rates of compulsive shoppers according to the Italian version of the CBS (5) cutoff score (42). In this sample, the CBS scores ranged from 14.35 to 55.35 (M = 30.60, SD = 9.61) and 12.9% (*N* = 31, M = 6; F = 25) obtained scores below the proposed cutoff for CB. Applying the cutoff score, we compared the compulsive and non-compulsive buyers (non-CBs) on variables of interest.

Chi-square test showed that compulsive buyers do not differ from non-CB on marital status, level of education, or income.

ANOVA analysis (see Table 1) showed that the compulsive buyers have a higher average of time spent shopping (hours per week) than the non-CB. In shopping experiences, the former are more influenced by advertising and have higher scores on CSE and FNE than the latter. However, the two groups did not differ in money spent per shopping trip and in the frequencies of credit card usage.

**Table 1:** **Means and ANOVA differences for compulsive buyers (CBs) and non-compulsive buyers (non-CBs)**.

	CB vs non-CB														
Measures	MCB (*N* = 31)	SD	M Non-CB (*N* = 209)	SD	F
Age	33.16	10.50	33.90	10.65	0.13
Media influence (1, 5)	3.52	0.85	2.65	0.88	26.55***
Credit card use (1, 5)	3.03	1.20	2.89	1.13	0.83
Time spent for week (hours)	3.39	2.11	2.05	1.61	17.04***
Money spent (Euro)	97.74	65.22	80.60	97.23	0.90
Contingent self-esteem	28.42	3.27	23.38	5.52	24.50***
Fear of negative evaluation	27.06	4.67	22.32	6.59	14.95***

***p < 0.001.

### Gender Differences in Buying Behaviors and Variables of Interest

Comparing male and female groups, the Chi-square test showed that women do not differ from men on marital status (χ^2^ = 2.59, df = 4, *p* < n.s.). With regard to level of education (χ^2^ = 33.52, df = 6, *p* < 0.000), the results showed that more females had a first cycle degree (35.3%) than males (17.7%). In addition, there were more males with only a primary school or a junior high certificate (17.2%) than females (1.2%). Women (50%) show a higher quota with a low income (χ^2^ = 20.88, df = 5, *p* < 0.001) than men (31.4%), and men are more represented in the range between 45,000 and 60,000 Euros, and more than 60,000 Euros (11.4 vs 1.8%).

As regards the most purchased products, women buy more clothing/shoes than men (61.2 vs 31.4%) and men are more interested in technological objects (22.9 vs 2.4%) and in sports equipment (18.6 vs 4.1%) than women (χ^2^ = 58.37, df = 9, *p* < 0.000).

ANOVA analysis comparing genders showed that women were younger than men, used credit cards less frequently, and spent less money per week in shopping activities.

The findings revealed that women have higher scores than men on the CSE and FNE scales; there was no significant difference between genders in the CBS scores and in the media influence on purchasing (see Table 2).

**Table 2:** **Means and ANOVA differences for gender and shopping behavior**.

	Gender				
Measures	M	SD	M women	M men	F
Age	33.8	10.61	32.19	37.71	14.16***
Media influence (1, 5)	2.76	0.92	2.78	2.71	0.60
Credit card use (1, 5)	2.86	1.14	2.74	3.14	6.28**
Time spent for week (hours)	2.22	1.74	2.30	2.04	0.29
Money spent (Euro)	82.81	93.78	67.47	120.07	16.62***
Compulsive buying scale	30.23	10.45	30.99	28.36	3.19
Contingent self-esteem	24.03	5.54	24.74	22.31	9.86**
Fear of negative evaluation	22.93	6.56	23.68	21.10	7.89*

*p < 0.05.

**p < 0.01.

***p < 0.001.

We then examined Pearson correlations between age, buying behaviors, and constructs of interest (CBS, CSE, and FNE) (see Table 3).

**Table 3:** **Correlations**.

	1	2	3	4	5	6	7	8
1. Age	–							
2. Media influence (1, 5)	0.06	–						
3. Credit card use (1, 5)	0.25**	0.24**	–					
4. Time spent for week (hours)	0.16*	0.27**	0.21**	–				
5. Money spent (Euro)	0.35**	0.26**	0.26**	0.20**	–			
6. Compulsive buying scale	0.01	0.40**	0.12	0.33**	0.14*	–		
7. Contingent self-esteem	−0.08	0.17**	−0.04	0.03	−0.01	0.50**	–	
8. Fear of negative evaluation	−0.27**	0.29**	0.09	0.10	−0.03	0.41**	0.57**	–

*p < 0.05.

**p < 0.01.

### Mediation Analysis

To analyze the hypothetical mediation of FNE on the effect of CSE on compulsive shopping for men and women (Hypothesis 3), two sets of regressions were computed. Multiple regression analyses showed that Set 1 (men) explained 0.37 while Set 2 (women) 0.29 of the variance in compulsive buying.

**Figure 2:**
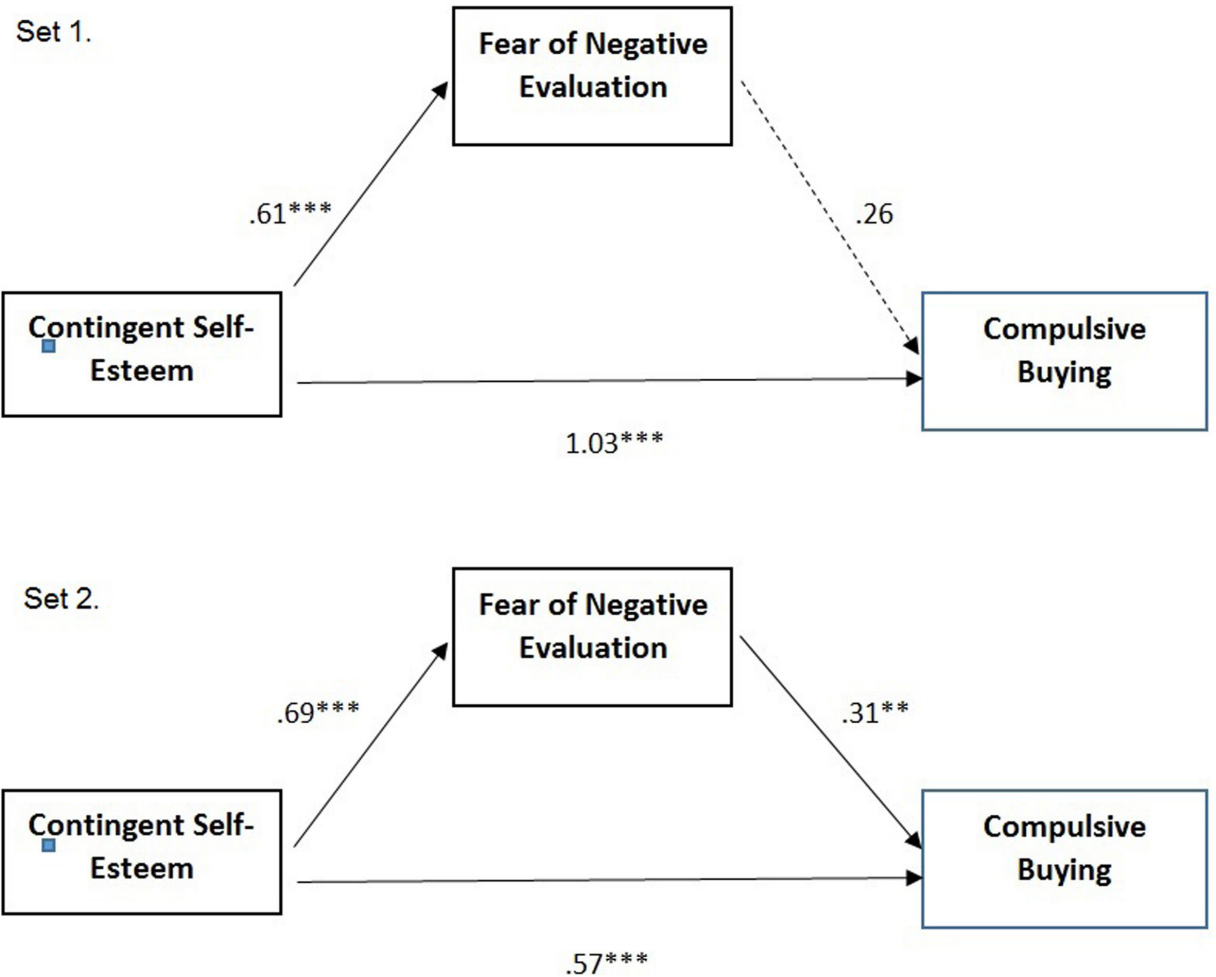
**Multiple regression analyses predicting compulsive buying for men (Set 1) and women (Set 2)**. Note: coefficients are unstandardized regression coefficients (*p < 0.05; **p < 0.01; ***p < 0.001).

As can be seen in Figure 2, the significant estimated paths were significant on both sets of regression except that of the mediating effect of FNE on compulsive buying for men. In both sets, CSE positively predicted FNE, whereas FNE positively predicted CB only for women.

Regarding the mediating effect of FNE, the effect of CSE on compulsive buying was partially mediated by FNE only for women (Set 2: indirect effect *B* = 0.2110, sobel test *z* = 2.28, *p* < 0.02; Set 1: indirect effect *B* = 0.16, sobel test *z* = 1.29, *p* = n.s.).

## Discussion

Preliminary results of our investigation into compulsive buying showed that the prevalence in our convenience sample agrees with data reported in the literature concerning Canada and European countries (5, 50, 53).

The time spent on shopping experiences is directly correlated to the CBS score. The result is consistent with McElroy et al. (1) who argued that the dimension of time spent on purchases is one of the proper diagnostic criteria of compulsive buying. Compulsive buyers spend more time on shopping trips than non-CB, but do not spend as much money; data support the hypothesis that it is the experience of browsing rather than purchasing itself that characterizes the disorder (41).

Moreover, in accordance with Maraz et al. (41), CB is not directly related to socioeconomic status or net income. In line with other studies [e.g., Ref. (5)], the findings did not reveal any relation between compulsive buying and credit card usage.

As regard the age influence, the findings reported that age is not related to compulsive shopping or to CSE while the FNE decrease with increased age. About this, Westenberg et al. (54) have argued that individual differences in social evaluation fear may be related to differential level of maturity.

As far as gender differences are concerned, our data partially confirmed the literature previously described suggesting that women scored higher on CSE and FNE scales than men (H1). In agreement with Mueller et al. (55), but nonetheless surprisingly, no significant difference was found between women and men in compulsive buying. Although the vast majority of people with compulsive buying problems have been estimated to be women [e.g., Ref. (56)], the gender differences reported by the literature may be artifactual and a bias could be due to the fact that women are more prone to admitting that they enjoy shopping than men (57).

Consistent with prior research [e.g., Ref. (6, 58, 59)], our findings suggest that appearance-based objects such as clothes and shoes are most frequently purchased by women. Such image-based objects strengthen feelings of self-worth by regulating emotions, gaining social approval, and expressing an idealized self (18).

However, men tend to buy electronic or hardware goods more than women and buying objects such as technological items could be a strategy to signal social status and to impress others also in men. Dittmar et al. (60) reported that men tend to impulsively buy instrumental and leisure items associated with independence and activity, while women tend to purchase symbolic items associated with appearance and emotional aspects of self (e.g., attractiveness) (61, 62). Choosing certain objects to buy can be gender-specific, but the motivation for purchases may be similar, for instance to impress others.

In addition, differently from the literature [e.g., Ref. (63)], we did not find any gender differences in the time spent on shopping trips. Instead, women spend less money and use credit cards less than men. It should be noted that in our sample, the men are on average older and have a higher annual income than women. Moreover, in accordance with the literature (64), male consumer characteristics are different from those of females and men are generally more willing “to take risks with money” than women. Some authors (65) argued that men who shop prefer to see themselves as fulfilling an instrumental need, rather than engaging in shopping *per se*. They bypass the female relational aspects of shopping experience, looking for bargains, and are more prone to spending in order to pursue business goals.

As hypothesized (H2), the survey findings revealed that CSE impacts on compulsive buying behavior in both genders. CSE proved to be a strong predictor of CB. Theories on self-esteem long held that the sense of self is largely built through social interaction with significant others. Indeed, feelings of self-esteem tend to result from the approval of others. For individuals who view approval as contingent to success, such as getting good grades, winning a game, or living up to standards of physical attractiveness, self-esteem becomes largely dependent on the others’ judgment (32). In agreement with Roberts et al. (28), our model suggested that individuals higher in CSE are more likely to become compulsive shoppers. Specifically, the mechanism underlying compulsive buying may be the process of “symbolic self-completion” (60). Some people need others to acknowledge that they possess a specific self-definition. To feel a sense of completion in his/her own self-definition, an individual must engage in behaviors related to symbols (e.g., possessing and wearing them) insofar as he/she convinces others, and consequently him/herself, which he/she owns the self-definition hoped for.

Some authors (66) have suggested that appearance-related items (e.g., clothes, shoes, and status symbol objects) are often used as symbols in the process of self-completion “because they show others who you are.” Some individuals may feel a chronic state of incompleteness in their self-definitions and may experience the urge to buy objects linked to their desired self-definitions despite the fact that they have purchased too much, resulting in serious problems for their life (60).

Instead, even if CSE predicts FNE in both genders, this variable appears to have no effect on compulsive buying for men. These data do not fully confirm the findings of Roberts et al. (28) and suggest that a certain level of CSE may lead women to consider compulsive shopping as an “antidote” for FNE whereas this is not true for men. Those high in CSE are more likely to buy compulsively, but this relationship is partially mediated by the FNE only in women (H3).

However, the absence of mediation between FNE and CB in men was unexpected. Men with high CSE are more likely to use shopping as a coping strategy but are less affected by the fear of external judgment, or are less prone to admitting it.

To explain this, in accordance with some authors [e.g., Ref. (67)], we assume that one of the most significant differences between men and women is the difference in their self-concept, compatible with gender stereotypes. In particular, males and females differ in regard to how much they define themselves as autonomous agents or they view themselves as connected with others (34). In Western societies, females can develop an “interdependent self-concept,” and conversely, males develop an “independent self-concept,” with reference to self-definitions of being “separated from others,” following “individualistic goals,” and being motivated “to show uniqueness by power over others” (68). Contrary to this, interdependent self-concept, more typical of females, refers to self-definitions such as “connection with others,” where “relationships are perceived as integral parts of one’s being” (34). Some authors argued that women who are more socially self-conscious suffer from greater self-discrepancies (49). Higgins (69) proposed that women who feel there are discrepancies between their actual attributes and their sense of how others think they ought to be also experience increased FNE and punishment, which may result in an unhealthy cycle of self-discrepancy and fear of evaluation (70). Conversely, males show a weaker sensitivity to the opinions of others (71).

One other explanation for the observed gender differences in the mediation role of FNE is the particular pressures faced by women to conform to societal standards. Women are held to more prescriptive and higher social standards compared to men (72), and violating these standards carries more threat of penalty and punishment for women than men (73). FNE may enhance the negative effects of failing to live up to one’s standards, particularly for women.

In summary, the models tested in the present study have contributed to the understanding of psychological processes underlying compulsive buying behavior; in specific, they explained how CSE and the fear of disapproval are involved in this behavioral addiction. The findings suggest that individuals who have a stable sense of worth, and do not depend on others’ evaluation, could theoretically have a barrier against the risk of developing such dysfunctional behavior. Conversely, CSE and FNE represent risk factors for problematic addictions, especially in women. Paradoxically, individuals who base their self-worth on contingencies continue to compare themselves with others, thus setting up a vicious circle of self-disapproval. Compulsive shopping can, therefore, appear as an illusory and counterproductive solution to these negative feelings.

### Limitations and Implications

Although this research expands the current knowledge on gender differences in the mechanisms underlying CB, such as CSE and FNE, it presents certain limitations.

The main limitation of the study is that its cross-sectional nature precludes the assessment of cause–effect relationships. That is, although the results suggest statistical predictive effects, these should be more properly tested in a longitudinal model.

Next, the external validity of our findings is limited by the sampling procedure. We used a convenience sample retrieved online that does not allow generalization of the results. The age of the participants is not homogeneous; as FNE is inversely related to age, it would be interesting to test if the mediation model changes for more homogeneous samples (e.g., late adolescence or elderly). Another limitation is the low male response rate. The problem of the low response of men in compulsive buying studies has been recognized in the literature: males are reluctant to respond to inquiries regarding shopping (18).

Furthermore, other psychopathological disorders have not been explored; first of all, depression is a dimension very often associated with pathological purchases that might affect differently men and women as a risk factor for compulsive buying (74). Moreover, comorbidity with other behavioral addictions should be investigated (75).

However, despite these limitations, the results of our study may also have implications for clinical intervention. Identifying predictors and mediators of CB is important for tailoring addiction prevention programs and for improving interventions more suited to individual psychological characteristics. Overall, the findings provide suggestions for future appropriate interventions focused on personality characteristics. These interventions should distinguish between different personality features, taking into account gender differences in CSE. Future research should also focus on men’s and women’s motivations to buy, which could predict or mediate individual vulnerability traits and compulsive buying.

## Ethics Statement

APA ethical standards were followed in the conduct of the study.

## Author Contributions

The author designed the study, conducted the statistical analysis, and wrote the manuscript.

## Conflict of Interest Statement

The author declares that the research was conducted in the absence of any commercial or financial relationships that could be construed as a potential conflict of interest. The reviewer, MN, and handling editor declared their shared affiliation, and the handling editor states that the process nevertheless met the standards of a fair and objective review.
